# Legume-grass mixtures increase forage yield by improving soil quality in different ecological regions of the Qinghai-Tibet Plateau

**DOI:** 10.3389/fpls.2023.1280771

**Published:** 2023-10-19

**Authors:** Feng Luo, Wenhui Liu, Wenbo Mi, Xiang Ma, KaiQiang Liu, Zeliang Ju, Wen Li

**Affiliations:** Key Laboratory of Superior Forage Germplasm in the Qinghai-Tibetan Plateau, Qinghai Academy of Animal Husbandry and Veterinary Sciences, Qinghai University, Xining, China

**Keywords:** forage mixtures, ecological regions, forage yield, soil enzyme activity, soil nitrogen fraction, soil microorganism, Qinghai-Tibet Plateau

## Abstract

**Introduction:**

Information on the relationship between soil quality and forage yield of legume-grass mixtures in different ecological regions can guide decision-making to achieve eco-friendly and sustainable pasture production. This study’s objective was to assess the effects of different cropping systems on soil physical properties, nitrogen fractions, enzyme activities, and forage yield and determine suitable legume-grass mixtures for different ecoregions.

**Methods:**

Oats (Avena sativa L.), forage peas (Pisum sativum L.), common vetch (Vicia sativa L.), and fava beans (Vicia faba L.) were grown in monocultures and mixtures (YS: oats and forage peas; YJ: oats and common vetch; YC: oats and fava beans) in three ecological regions (HZ: Huangshui Valley; GN: Sanjiangyuan District; MY: Qilian Mountains Basin) in a split-plot design.

**Results:**

The results showed that the forage yield decreased with increasing altitude, with an order of GN (3203 m a.s.l.; YH 8.89 t·ha-1) < HZ (2661 m; YH 9.38 t·ha-1) < MY (2513m; YH 9.78 t·ha-1). Meanwhile, the forage yield was higher for mixed crops than for single crops in all ecological regions. In the 0-10 cm soil layer, the contents of total nitrogen (TN), microbial biomass nitrogen (MBN), soil organic matter (SOM), soluble organic nitrogen (SON), urease (UE), nitrate reductase (NR), sucrase (SC), and bacterial community alpha diversity, as well as relative abundance of dominant bacteria, were higher for mixed crops than for oats unicast. In addition, soil physical properties, nitrogen fractions, and enzyme activities varied in a wider range in the 0-10 cm soil layer than in the 10-20 cm layer, with larger values in the surface layer than in the subsurface layer. MBN, SON, UE, SC and catalase (CAT) were significantly and positively correlated with forage yield (P < 0.05). Ammonium nitrogen (ANN), nitrate nitrogen (NN), SOM and cropping systems (R) were significantly and positively correlated with Shannon and bacterial community (P < 0.05). The highest yields in the three ecological regions were 13.00 t·ha-1 for YS in MY, 10.59 t·ha-1 for YC in GN, and 10.63 t·ha-1 for YS in HZ.

**Discussion:**

We recommend planting oats and forage peas in the Qilian Mountains Basin, oats and fava beans in the Sanjiangyuan District, and oats and forage peas in Huangshui valley. Our results provide new insights into eco-friendly, sustainable, and cost-effective forage production in the Qinghai Alpine Region in China.

## 1 Introduction

Due to the rapid development of the global economy and the growing population, the dietary structure of the population has changed, and the demand for high-protein meat products is increasing, and the relationship is likely to get even closer in the future. This relationship will strengthen in the future. In addition, the livestock industry faces challenges as the demand for forage intensifies (Hisham et al., 2022). A consistent forage supply is critical for grass-based livestock husbandry and food security. Natural grasslands are the primary forage source for livestock (Neto et al., 2021). However, human activities and other factors have resulted in grassland degradation in recent years due to climate change, reducing ecological service functions (Cao et al., 2019). Planting forage is critical for grassland recovery and feeding livestock (Dong et al., 2010). Forage grass cultivation is a crucial aspect of the livestock industry and has seasonal and regional characteristics (Tahir et al., 2023). However, insufficient soil fertility and excessive nitrogen fertilizer inputs aimed at increasing forage yields have increased the cost of agricultural production (Moorhead et al., 2013). Environmental problems have occurred due to the negative impacts of nitrogen fertilizers on the nitrogen cycle and nitrogen balance, hindering sustainable agriculture (Song et al., 2020). Thus, planting forages is required in livestock operations to promote its efficient and effective development and to repair the ecological barrier. Forage production affects the environment because it requires resources, including land, energy, water, and labor (Peeters and Parente, 2006).

Legume–grass mixtures are planted to increase forage biomass (Tilman et al., 2002), nutritional quality, resource utilization, productivity (Loreau and Hector, 2001; Loreau et al., 2001) and soil fertility to reduce pollution of grassland agro-ecosystems by inorganic fertilizers (Heijden and Horton, 2009; Frankow-Lindberg and Dahlin, 2013). Meanwhile, which can balanced feed for livestock and sustain the soil nutrient balance with minimal environmental impact (Liu et al., 2022; Tahir et al., 2022). Additionally, mixtures of two to four species may provide optimum plant diversity and limit seedling competition compared to more complex mixtures (Foster et al., 2014).Mixed grasslands improve N mineralization and utilization by influencing the soil carbon/nitrogen balance. They increase organic matter inputs and the abundance of beneficial soil microorganisms (Fornara and Tilman, 2008; Fornara et al., 2009; Deyn et al., 2011). As a result, microbial enzyme activity and mineralization of nutrients are improved, and yields are increased (Sun et al., 2015). However, the soil microbiome is sensitive to environmental and crop influences (Brockett et al., 2012). Therefore, it is necessary to analyze soil nitrogen fractions, enzyme activities and microbial community in mixed legume-grass pastures in different ecological zones and assess forage productivity to improve livestock production.

The Qinghai–Tibet Plateau (QTP) is known as the “roof of the world” and the “third pole” due to its average elevation of 4,000 m. Unique climatic conditions and geographical location have resulted in an alpine grassland ecosystem (Wei et al., 2014). The alpine meadows of the QTP, the largest natural grassland in China, cover an area of approximately 2.27×10^6^ hm^2^. They represent an ecological barrier, are critical forage sources, and provide essential ecological functions and social services for the inhabitants (Hu et al., 2021). The grasslands have been significantly degraded in recent years due to overgrazing, poor management,and global climate change (Harris, 2010; Ren et al., 2014; Xue et al., 2017), reducing ecosystem services and exacerbating the imbalance between forage supply and grass-fed animal feed demand (Cao et al., 2011; Niu et al., 2019). However, the forage types that can grow in this environment are limited due to the cold and arid climate and insufficient soil fertility; thus, a lack of forage is the main limiting factor affecting the development of the livestock industry in this region. Oats is widely planted and nitrogen fertilizers are applied in the region to ensure sufficient forage. However, high-protein forage is lacking, and the high nitrogen inputs cause environmental pollution. Therefore, measures are required to replace nitrogen fertilizer inputs, such as planting leguminous crops that fix nitrogen. Legume-oat mixtures are used to increase forage yields and reduce environmental pollution. In addition, many microclimates exist in the alpine region due to large differences in altitude and climate. The optimal mixed crops suitable for different ecological zones in the alpine region have not been determined to date. However, there is an urgent need to investigate the forage yields of mixed crops in different alpine ecological zones to improve forage yields and mitigate the negative impacts on the environment.

Consequently, this study investigates the effects of different forage crops on soil physical properties, soil nitrogen fraction, soil enzyme activities, and yield in different alpine ecological regions. The results of this study provide guidance for forage production in Qinghai, China, using mixed crops that can mitigate negative environmental impacts and promote sustainable agricultural systems.

## 2 Materials and methods

### 2.1 Study site

**Figure 1:**
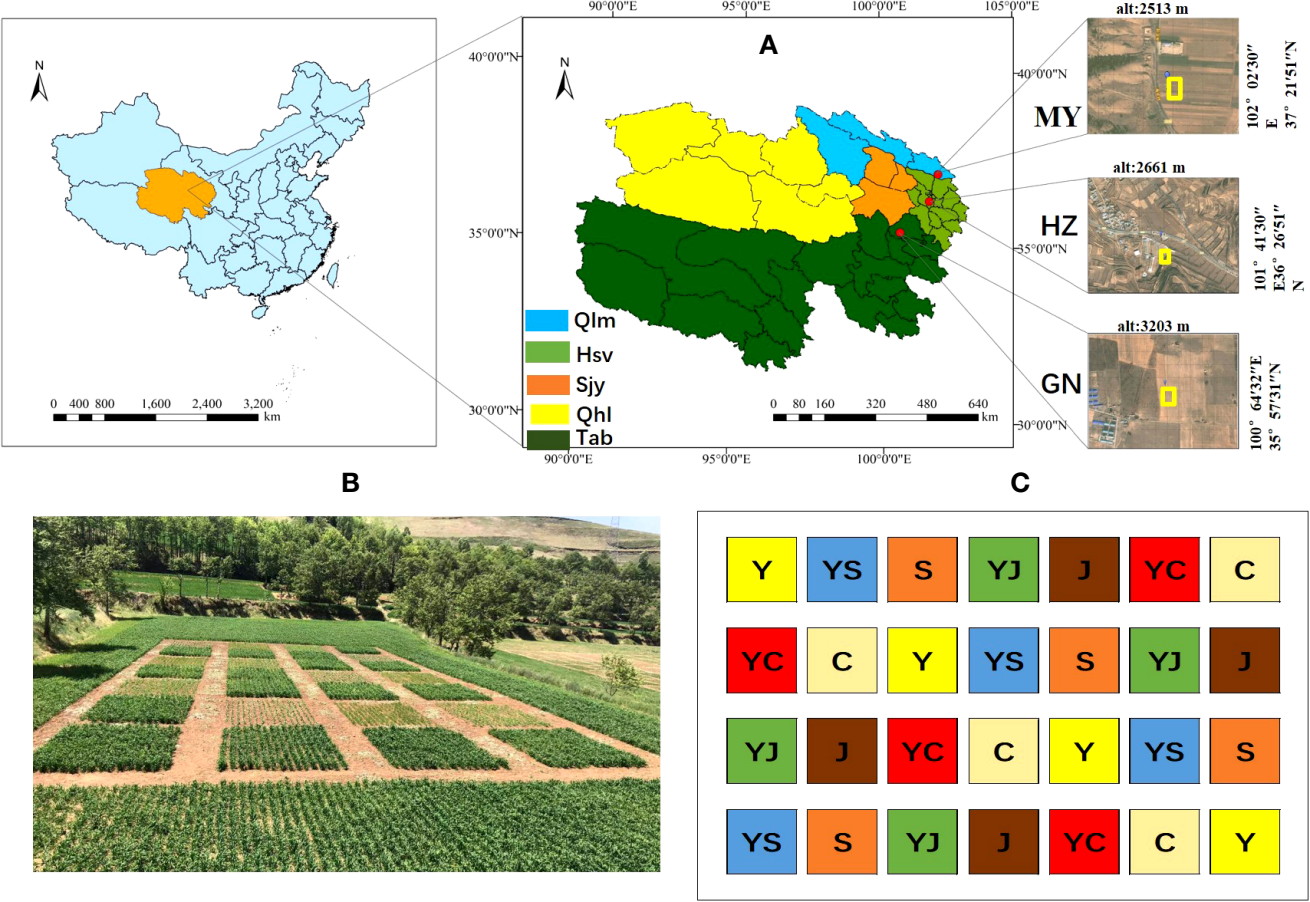
Study area and cropping map. **(A)** Study area information. Ecological regions (Qlm, Qilian Mountains Basin; Hsv, Huangshui Valley; Sjy, Sanjiangyuan District; Qhl, Qinghai Lake District; Tab, Tsaidam Basin). Red, study area (HZ, Huangshui Valley; GN, Sanjiangyuan District; MY, Qilian Mountain Basin). **(B, C)** overview of one of the experimental fields and cropping systems (Y, oats unicast; YS, oats and forage peas mixed sowing; S, forage peas unicast; YJ, oats and common vetch mixed sowing; J, common vetch unicast; YC, oats and fava beans mixed sowing; C, fava beans unicast) The same as below.

The experiment was conducted at one planting site each in three ecological regions of Qinghai Province (**Figure 1**):

Huangzhong County (HZ) in Huangshui Valley: the site is located in the village of Tumen Pass Township Garur (101° 41 ′ 30 ″ E, 36° 26 ′ 51 ″ N). The elevation is 2661 m, the average annual temperature is 5.3°C, and the average annual precipitation is 490 mm. The climate is cold and humid, and there is no frost-free period. Buckwheat was planted previously at the site.

Guinan County (GN) in the Sanjiangyuan District: the site is located in Sendo Town (100°64′32″ E, 35°57′31″ N). The elevation is 3203 m, the average annual temperature is 3.2°C, and the average annual precipitation is 403 mm. The climate is cold and humid, and there is no frost-free period. Rapeseed was planted previously at the site.

Menyuan County (MY) in Qilian Mountain Basin: The site is located in Xianmi Township (102°02′30″ E, 37°21′51″ N). The elevation is 2513 m, the average annual temperature is 4.2°C, and the average annual precipitation is 518 mm. The climate is cold and humid, and there is no frost-free period. Astragalus was planted previously at the site. The soil physical and chemical properties of the study sites are listed in **Table 1**.

**Table 1:** Soil physicochemistry of the study site.

Ecological region profile			
Basic information	Huangshui Valley	Sanjiangyuan Area	Qilian Mountain Basin
Total nitrogen (g·kg^-1^)	2.2	2.4	3.1
Total phosphorus (g·kg^-1^)	2.5	1.7	2.0
Total potassium (g·kg^-1^)	24.4	18.1	21.1
Alkali-hydrolyzed nitrogen (mg·kg^-1^)	120.00	105.0	124.0
Available phosphorus (mg·kg^-1^)	27.6	20.3	26.1
Available potassium (mg·kg^-1^)	290.0	244.0	258.0
Organic matter (g·kg^-1^)	34.4	34.4	50.1
Soil texture	calcium chestnut soil	clay loam	black calcium soil

### 2.2 Experimental design

A randomized block design was used, with three plots of mixed crops (oats/forage peas, oat/common vetch, and oats/fava beans) and four plots of monocultures (oats, common vetch, fava beans, and forage peas, resulting in seven treatments. We used four replicates for each treatment, for a total of 28 plots, each of which was 4 m × 5 m. The crops (Qinghai 444 oats, Qingjian No.1 forage peas, Ximu No.324 common vetch, and green fava beans) were provided by the Qinghai Academy of Animal Husbandry and Veterinary Science. The No.22 fava beans were provided by the Qinghai Provincial Academy of Animal Husbandry and Veterinary Science.

Based on the local climate and planting dates, the Huangzhong, Menyuan, and Guinan test sites were sown on 6 May, 21 May, and 29 May 2022, respectively. Before sowing, a base fertilizer consisting of urea (75 kg·ha^-1^) and calcium superphosphate (150 kg·ha^-1^) was applied. Sixteen rows were sown in strips with a row spacing of 25 cm and a depth of 6-8 cm. The fava beans were sown individually with a spacing of 10 cm between the seeds and a row spacing of 25 cm. The other crops were sown in strips with the same row spacing. It has been shown that in the alpine region of Qinghai-Tibet, the optimum mixing ratios of oats with forage peas, oats and fava beans, and oats and common vetch are 6:4, 7:3, 6:4 (Xiang, 2022). The sowing amount is listed in **Table 2**. The field management was consistent with other crops. Manual weed control was performed twice, and the plots of each species were harvested at the same time.

**Table 2:** Planting systems and sowing rate.

cropping systems	Crop	Seeding quantity/g·m^-2^
Y/S	Oat	13.50
	Forage peas	4.45
Y/J	Oat	15.8
	Common vetch	6.61
Y/C	oat	13.50
	Fava bean	6.61
Y	Oat	22.50
S	Forage peas	11.16
J	Common vetch	12.00
C	Fava bean	16.53

Y, oats unicast; YS, oats and forage peas mixed sowing; S, forage peas unicast; YJ, oats and common vetch mixed sowing; J, common vetch unicast; YC, oats and fava beans mixed sowing; C, fava beans unicast.

### 2.3 Sampling and measurements

Due to the large differences in elevation and climate between the three test sites, the crops had different growth and harvest periods. The HZ, MY, and GN test sites were forage harvest and soil samples collected on September 6, September 20, and September 28, 2022 (when the oat plants were in the milky stage and the beans were in the podding stage), respectively.

#### 2.3.1 Biomass yield

The aboveground portion of all plants in each plot was mowed (stubble height of 3-5 cm) flush to the ground (excluding the 50 cm portion of the side rows and the two sides) and weighed to obtain the fresh forage yield. Fresh samples (1000 g) were obtained from each test plot, placed into air-drying bags, and brought back to the laboratory. The material was placed in an oven at 105°C for 30 min, and the temperature was reduced to 70°C until a constant weight was achieved. The fresh-dry ratio was calculated and converted into the yield per hectare.

#### 2.3.2 Soil sampling

Soil near the crop roots was collected from 28 test plots using an auger. We randomly collected 5 soil sub-samples (0-10,10–20 cm depth) from each experimental plot and integrated them into one soil sample to reduce errors due to soil heterogeneity (Edwards, 2010). This approach is commonly used in soil microbiology and for determining the physico-chemical properties of soil (Huhe et al., 2017). The soil sample was sieved through a 2-mm mesh to homogenize the sample and eliminate stones, roots, and plant residues. Each sample was divided into 2 aliquots. One was stored at an ambient temperature to dry naturally for use in the chemical assay, and the other was cryopreserved at -80°C for the subsequent microbial biomass analysis.

#### 2.3.2 Soil properties

The soil pH was measured with a digital pH meter (Seven2Go, Mettler-Toledo Instruments Co., Ltd, Shanghai, China) in a 1:2.5 soil/water suspension after shaking at 250 rpm for 5 min. The soil physicochemical properties were determined following the “Soil Sampling and Methods of Analysis” (Crepin and Johnson, 1993). The soil bulk density (BD) was measured with the cutting ring (100cm^3^) method. Total soil porosity (TP) was obtained by coefficient conversion. Soil organic matter (SOM) was measured using the K_2_Cr_2_O_7_ redox titration method. Total nitrogen (TN) in the soil was determined by Kjeldahl digestion. Soil ammonium nitrogen (ANN) was determined by the indophenol blue colorimetric method. Nitrate nitrogen (NN) in the soil was assessed using the phenol disulfonic acid colorimetric method. The soil SON was extracted by hot water (70°C) using the modified methods described by Chen (Chen et al., 2005). Briefly, 10 g (dry weight equivalent) of fresh soil was mixed with 40 mL of distilled water in a Falcon tube, and the tube was placed in a hot water bath for 18 h at 70°C. The tubes were subsequently shaken for 5 min on an end-to-end shaker and filtered through Whatman 42 paper followed by a 0.45 mm filter membrane. The concentrations of NH_4_^+^-N, NO_3_^−^-N and NO_2_^−^-N in the extracts were measured using a LACHAT QuickChem Au-tomated Ion Analyser (QuikChem Method 10-107-06-04-D for NH_4_^+^ -N and Quik Chem Method 12-107-04-1-B for NO_3_^−^-N). The soluble inor-ganic N (SIN) was calculated as the sum of the NH_4_^+^-N, NO_2_^−^-N and NO_3_^−^-N in the extracts. The total soluble N (TSN) in the extracts was analyzed by the high temperature catalytic oxidation method using a SHIMADZU TOC analyzer (fitted with a TN unit) as described by Chen (Chen et al., 2005). The SON was calculated as the difference between the TSN and SIN. Soil microbial biomass nitrogen (MBN) was determined by leaching using chloroform fumigation (Joergensen, 1996).

#### 2.3.3 Soil enzyme activities

The nitrate reductase (NR) activity was measured following the method described by (Abdelmagid and Tabatabai, 1987) using KNO_3_ as the substrate. It was calculated after NO_3_^−^ reduction following 24^-^h incubation at 25°C. The urease (UE) activity was assessed by incubating 5 g of the fresh soil sample for 2 h at 37°C with 2.5 mL of a 0.08 M urea solution. The NH_4_^+^ content was determined using a spectrophotometer at 690 nm (Kandeler and Gerber, 1988). The soil sucrase (SC) activity was evaluated by determining the glucose discharge from an 8% sucrose solution following 24 h of incubation at 37°C (Chen et al., 2010). The alkaline protease (ALPT) activity was analyzed using the ninhydrin colorimetric method (Liu et al., 2015). The potassium permanganate titration method was used to assess the catalase (CAT) activity (Wang et al., 2021).

#### 2.3.4 High throughput sequencing of soil samples

Sequencing was done by the Wekemo Biotechnology Co., Ltd (Guangzhou, China). Soil DNA was extracted from 0.25 g using the HiPure Soil DNA Kit (Wekemo, Guangzhou, China) according to the manufacturer’s protocol. Twenty DNA extracts were obtained and then stored at -80°C for further analyses. DNA degradation and impurity were detected by 1% agarose gel electrophoresis, DNA purity was assessed by NanoDrop 2000 UV-vis Spectrophotometer (Thermo Fisher Scientific, Wilmington, DE, USA), and DNA concentration was determined on a Qubit 3.0 Flurometer (Thermo Fisher Scientific). The hypervariable V3-V4 region of the bacterial 16S rRNA gene was amplified with primer pairs 341F (5’-CCTACGGGNGGCWGCAG-3’) and 806R (5’-GGACTACHVGGGTWTCTAAT-3’) by an ABI GeneAmp^®^ 9700 PCR thermocycler (ABI, Foster City, CA, USA). HiSeq sequencing and PE250 sequencing strategy were used.

### 2.4 Statistical analysis

All reported results are the mean of three replicates. The data were analyzed using SPSS software (version 28.0: IBM Corp., Armonk, NY, USA). The effects of cropping systems and cropping regions on soil’s physical properties, nitrogen fractions, enzyme activity, and forage yield were analyzed using a two-way analysis of variance with Duncan’s multiple-range test. A value of *p* < 0.05 was considered statistically significant, and a value of *p* < 0.01 was considered very significant. Origin 2021 (OriginLab, Massachusetts) software was used to draw graphs. The tables and graphics were created using Excel 2019 and GraphPad Prism 8, respectively. Soil microbial community a-diversity was assessed using chao1, observed species number, shannon indices. soil microbial b-diversity were analyzed by nonmetric multidimensional scaling analysis (NMDS). The relationships between the variables— soil physical properties, nitrogen fractions, enzyme activities, soil microbial diversity, and forage yield—were determined by calculating Mantel test and Pearson correlation coefficients. The structural equation model (SEM) was implemented using R. Statistical analyses and drawings used R 4.3.1 for Windows and the “vegan”, “ggplot2”, “ggcor”, “dplyr”, “piecewiseSEM” package.

## 3 Results

### 3.1 Forage yield

The influences of the cropping systems on the forage yield in different ecological regions are listed in **Table 3**. The results showed that the ecological region, cropping system, and their interaction had significant effects on the forage yield (*P* < 0.01). However, the difference in the mean of forage yield in different ecological regions were not significant. The ranking of the mean values of forage yield in different cropping systems was mixed crops > oats > legumes. The forage yield of YS and YJ in HZ was significantly (*P* < 0.05) higher than that of Y (23.17%, 16.11%). The forage yield of YS and YC in GN was significantly (*P* < 0.05) higher than that of Y (22.51%, 22.85%). The yield of YS, YC in MY was significantly (*P* < 0.05) higher than that of Y (48.57%, 17.37%).

**Table 3:** Forage yield of different planting patterns in each ecological region.

Treatment	District			
	HZ	GN	MY	Average
Y	8.63 ± 0.32Ad	8.62 ± 0.39Bb	8.75 ± 0.2Ac	8.66 ± 0.32cd
YS	10.63 ± 0.56Aab	10.56 ± 1.61Ba	13.00 ± 0.49Aa	11.40 ± 1.53a
S	7.33 ± 0.62Acd	8.09 ± 0.44Ab	9.45 ± 0.29Abc	8.29 ± 1de
YJ	10.02 ± 0.21Abc	9.34 ± 0.24Aab	9.66 ± 0.38Bbc	9.68 ± 0.4ab
J	9.84 ± 0.38Acd	5.41 ± 0.92Bc	7.40 ± 0.24Cd	7.55 ± 1.9e
YC	9.83 ± 0.29Bcd	10.59 ± 0.2Ca	10.27 ± 0.59Ab	10.23 ± 0.5b
C	9.4 ± 0.52Acd	9.6 ± 0.41Bab	9.92 ± 0.59Ab	9.64 ± 0.56ab
Average	9.38 ± 1.08A	8.89 ± 1.78A	9.78 ± 1.69A	
F	District	8.81**		
	Treatment	31.45**		
	D*T	7.28**		

HZ, Huangshui Valley; GN, Sanjiangyuan District; MY, Qilian Mountain Basin; Y, oats unicast; YS, oats and forage peas mixed sowing; S, forage peas unicast; YJ, oats and common vetch mixed sowing; J, common vetch unicast; YC, oats and fava beans mixed sowing; C, fava beans unicast.

* and ** represent significant differences at the 0.05 and 0.01 level, respectively.

Lowercase letters represent the significant difference within the same ecological regions under different cropping systems, while uppercase letters indicate the significant difference within different ecological regions under the same cropping systems. Significance was employed at 0.05.

### 3.2 Soil physical properties

**Figure 2:**
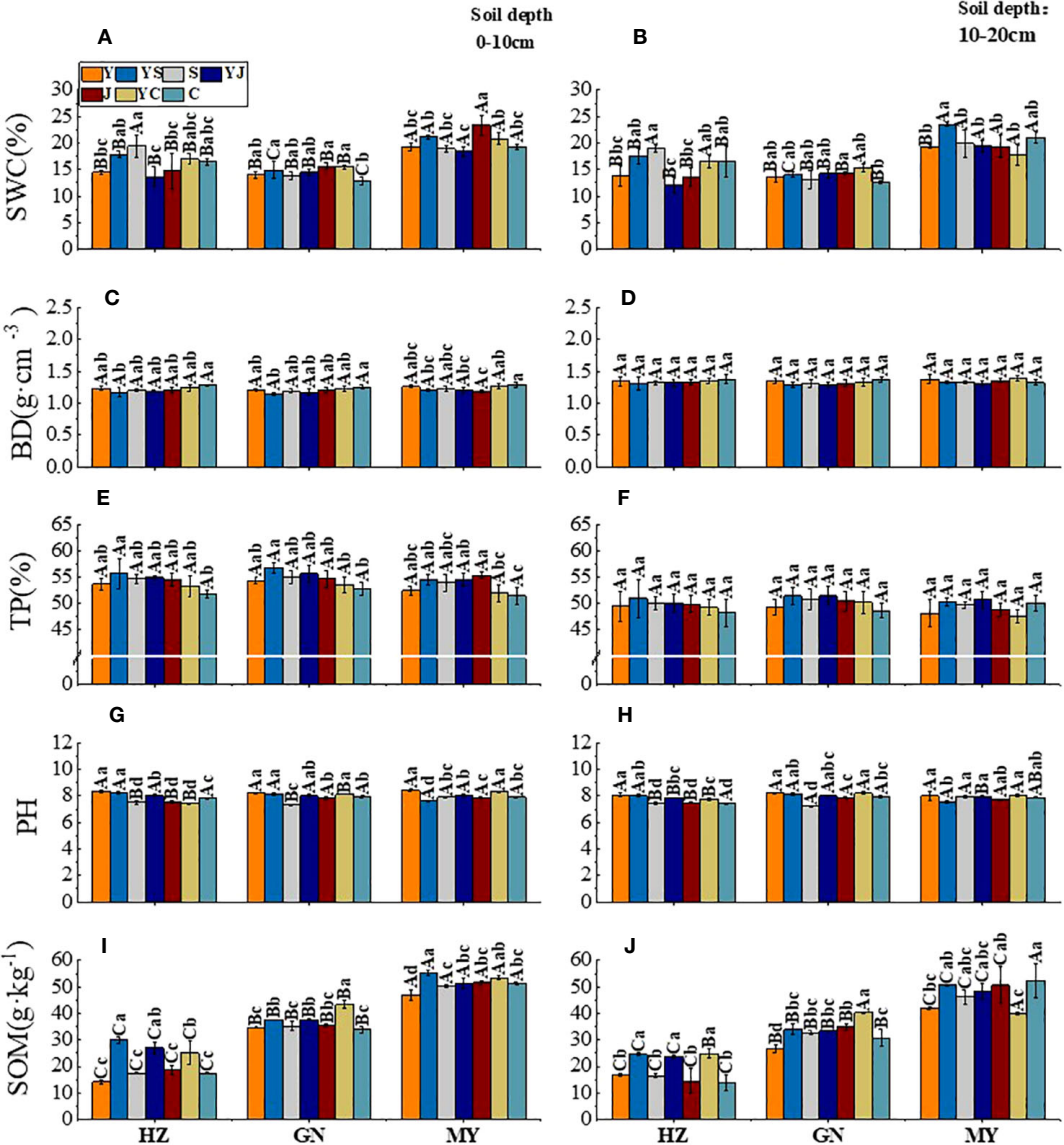
Effect of cropping systems on soil physical properties in different ecological regions. Ecological regions (HZ, Huangshui Valley; GN, Sanjiangyuan District; MY, Qilian Mountain Basin). Planting systems (Y, oat unicast; YS, oat and forage peas mixed sowing; S, forage peas unicast; YJ, oat and common vetch mixed sowing; J, common vetch unicast; YC, oat and fava beans mixed sowing; C, fava beans) **(A, B)** soil water content; **(C, D)** soil bulk density; **(E, F)**, soil porosity; **(G, H)** soil pH; **(I, J)** soil organic matter; **(A, C, E, G, I)** 0-10cm soil layer. **(B, D, F, H, G)** 10-20cm soil layer. The bars show the standard errors. Lowercase letters represent the significant difference within the same ecological regions under different cropping systems, while uppercase letters indicate the significant difference within different ecological regions under the same cropping systems. Significance was employed at 0.05. The same as below.

The effects of the cropping systems on the soil physical properties in the ecological regions are listed in **Table 4**. In the 0-10 cm soil layer, the cropping system and ecological region significantly influenced the SWC, BD, and TP (*P* < 0.01), and the interaction between the cropping system and the ecological region was significant (*P* < 0.01). In the 10-20 cm soil layer, the cropping system and ecological region significantly influenced the TP and SOM (*P* < 0.01), and the interaction between the cropping system and the ecological region was also significantly affected TP and SOM (*P* < 0.01). The soil’s physical properties showed a decreasing trend with the depth for different cropping systems and in different ecological regions. In addition, SOM and TP were higher, and the BD and pH were lower in mixed crops than in monocultures (**Figure 2**).

**Table 4:** Soil physicochemical variance analysis of cropping systems in different ecological regions.

Soil depth (cm)	Source of variation	SWC (%)	BD (g·cm ^-3^)	TP (%)	PH	SOM (g.kg^-1^)
0-10	District	76.413**	13.837**	13.473**	66.019**	2.881
	Treatment	4.227**	79.225**	28.444**	3.324	5.418**
	D * T	3.101**	6.006**	2.961**	3.054*	0.269
10-20	District	1.381	0.987	1381.473**	2.869	362.719**
	Treatment	1.005	1.206	32.396**	5.411**	5.589**
	D * T	0.306	0.303	6.655**	0.268	5.575**

SWC, soil water content; BD, soil bulk density; TP, total soil porosity; PH, soil acidity and alkalinity; SOM, soil organic matter. * and ** represent significant differences at the 0.05 and 0.01 level, respectively.

In the 0-10 cm soil layer, the SWC was higher in the legume monocultures (S/J/C) than in the other cropping systems in different ecological regions (**Figure 2A**). There was no significant difference between the BD and TP between the ecological regions. In contrast, the TP was higher for mixed crops than for legume monocultures in all ecological regions (except for the YS cropping systems in MY) (**Figure 2C, E**). The ranking of the pH values was oats > mixed crops > legumes (except for the YS mixed cropping system in MY) (**Figure 2G**). The ranking of SOM content in ecological regions was MY>GN>HZ. All cropping systems showed the same ranking based on the SOM: mixed crops > legumes > oats (except for the YS cropping systems in MY) (**Figure 2I**). In the 10-20 cm soil layer, the SWC was significantly higher in MY than in the other ecological regions, but the SWC was not significantly different in different cropping systems in the same ecological region (**Figure 2B**). There was no significant difference in the BD and TP between different cropping systems and ecological regions (**Figure 2D/F**). Although there were significant (*P* < 0.05) differences in the pH in different ecological regions, there was no significant difference in the pH between the cropping systems (**Figure 2H**). The highest SOM occurred in YS, and the improvement rate compared with the oat monoculture was GN (51.43%) > HZ (4.56%) > MY (2.05%) (**Figure 2G**).

### 3.3 Soil nitrogen components

**Figure 3:**
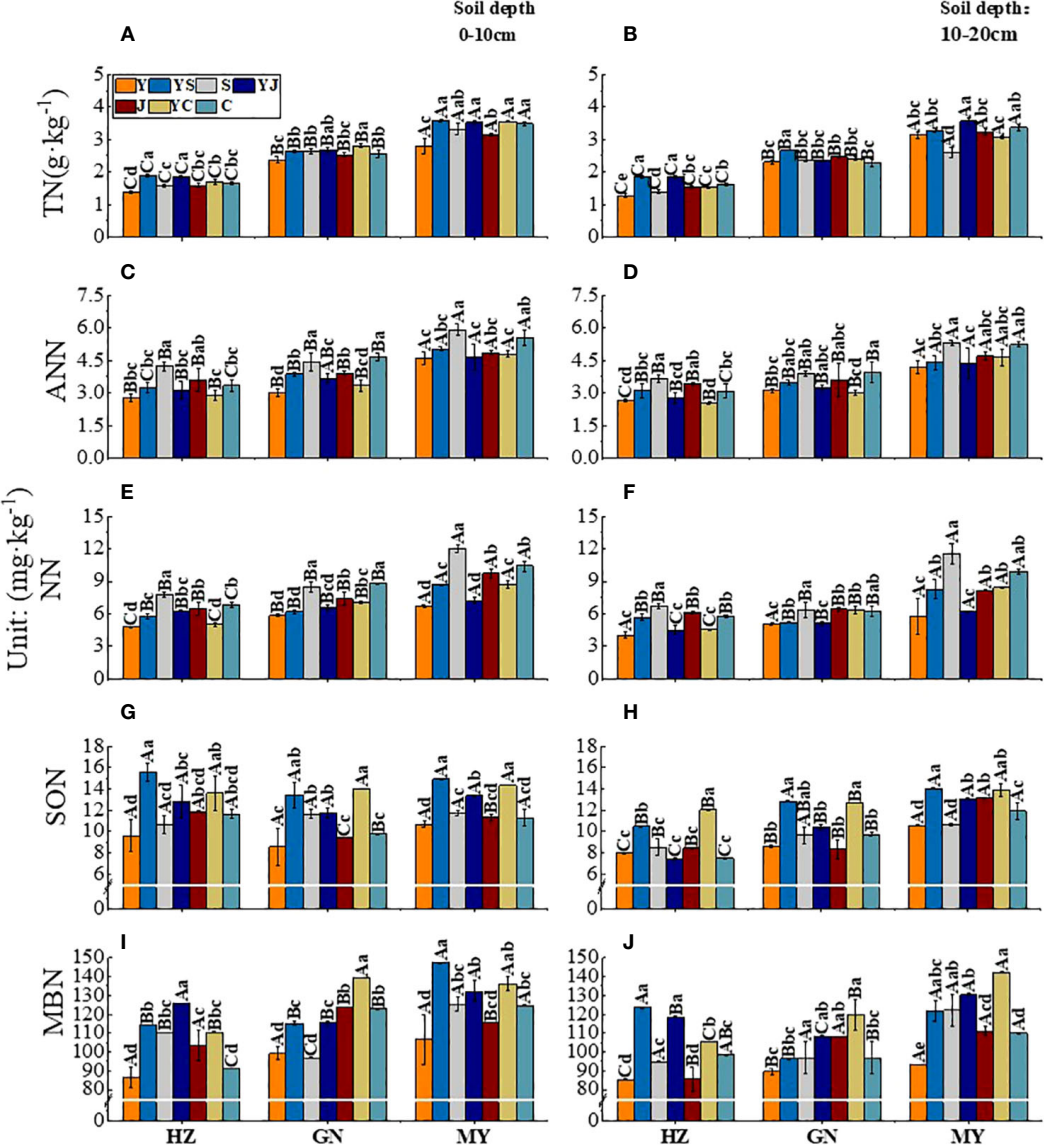
Effect of cropping systems on soil physical properties in different ecological regions. **(A, B)** soil total nitrogen; **(C, D)** Soil ammonium nitrogen; **(E, F)**, soil nitrate nitrogen; **(G, H)** soil soluble organic nitrogen; **(I, J)** soil microbial nitrogen; **(A, C, E, G, I)** 0-10cm soil layer. **(B, D, F, H, G)** 10-20cm soil layer. The bars show the standard errors. Lowercase letters represent the significant difference within the same ecological regions under different cropping systems, while uppercase letters indicate the significant difference within different ecological regions under the same cropping systems. Significance was employed at 0.05.

The influences of the cropping system on the soil nitrogen components in different ecological regions are listed in **Table 5**. In the 0-10 cm and 10-20 cm soil layers, the TN/ANN/NN/SON/MBN contents were substantially affected by the cropping system (*P* < 0.01), ecological region (*P* < 0.01), and their interaction (*P* < 0.01). However, the ANN/SON contents were not significantly affected by the interaction of the cropping system and ecological region in the 0-10 cm soil layer, and the ANN content was not significantly affected by the interaction between the cropping system and the ecological region in the 10-20 cm soil layer. There were significant differences in the soil nitrogen components in different ecological regions (*P* < 0.01). The contents of TN/AN/NN/SON/MBN decreased significantly with the soil depth in different cropping systems in different ecological regions (*P* < 0.01). The variation in the soil nitrogen components was higher in the 0–10 cm soil layer than in the 10–20 cm soil layer for all cropping systems in different ecological regions. In addition, the ranking of the SON content in all ecological regions was MY>GN>HZ, except in the 0-10 cm layer (MY>HZ>GN) (**Figure 3**).

**Table 5:** Variance analysis of soil nitrogen components in different cropping systems in different alpine ecological regions.

Soil depth (cm)	Source of variation	TN (g·kg^-1^)	ANN (mg·kg^-1^)	NN (mg·kg^-1^)	SON (mg·kg^-1^)	MBN (mg·kg^-1^)
0-10	District	1404.44**	136.247**	328.53**	9.33**	93.74**
	Treatment	29.71**	17.059*	99.40**	28.14**	43.44**
	D * T	3.73**	1.29	10.00**	1.35	13.95**
10-20	District	1770.15**	105.78**	134.35**	214.4**	72.91**
	Treatment	36.39**	8.56**	28.21**	63.21**	51.58**
	D * T	12.38**	0.77	5.93**	9.31**	12.75**

TN, soil total nitrogen; ANN, soil ammonium nitrogen; NN, soil nitrate nitrogen; SON, soil soluble organic nitrogen; MBN, soil microbial nitrogen. * and ** represent significant differences at the 0.05 and 0.01 level, respectively.

In the 0-10 cm soil layer, the ranking of the TN content in different ecological regions of all cropping systems was mixed crops > legumes > oats. The cropping systems with the highest soil TN contents in the three ecological regions were YS in MY, YS in HZ, and YC in GN (**Figure 3A**). The order of the ANN content in different ecological regions of all cropping systems was beans > mixed crops > oats. The cropping systems with the highest soil ANN contents were J (5.91 mg·kg^-1^) in MY, C (4.65 mg·kg^-1^) in GN, and S (4.23 mg·kg^-1^) in HZ. (**Figure 3C**). The mixed crops with the highest soil NN contents were YJ (HZ), YC (GN), and YC (MY). The soil NN content was 5.22%, 22.20%, and 30.45% higher, respectively than that of the oat monoculture (*P* < 0.05) (**Figure 3E**). The order of the SON content in different ecological regions of all cropping systems was mixed crops > legumes > oats (**Figure 3G**). The soil MBN ranged from 86.18 to 147.33 mg·kg^-1^ in the three ecological regions, with the highest values in mixed crops, followed by legumes and oats (**Figure 3I**).

In the 10-20 cm soil layer, the soil TN content was higher for mixed crops than for monocultures, except in MY. The soil TN content was significantly higher in YS than in the other cropping systems (*P* < 0.05) (**Figure 3B**). The soil ANN content was the highest in the legume monoculture in all ecological regions unicast, and the order of the three legumes was C > S > J (**Figure 3D**). There was a significant difference in the NN content between different ecological regions (*P* < 0.05). The soil NN content was significantly higher in the S cropping systems than in the other cropping systems. It was the lowest in the Y cropping system (**Figure 3F**). The legume monoculture had the lowest SON content, except in MY. The cropping systems with the highest SON contents in the ecoregions were YC or YS. The contents were significantly (P < 0.05) higher than those in the other cropping systems (**Figure 3H**). In MY, the MBN of YS and YJ were 12.65 and 118.60 mg·kg^-1^, respectively, significantly higher than those of the other cropping systems (*P* < 0.05) (**Figure 3J**).

### 3.4 Soil enzyme activity

**Figure 4:**
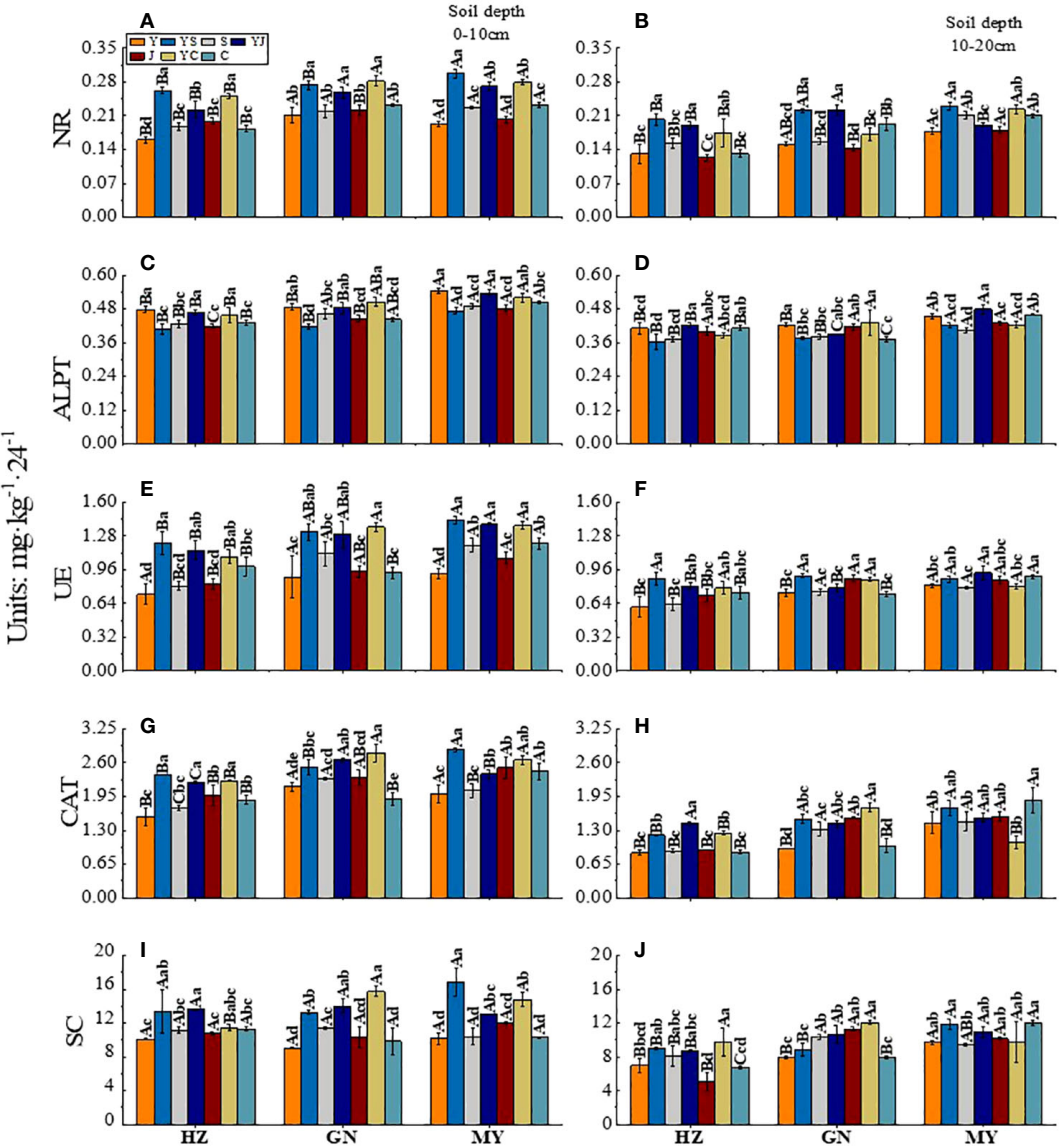
Effect of cropping systems on soil physical properties in different ecological regions. **(A, B)** urease; **(C, D)** alkaline protease; **(E, F)**, nitrate reductase; **(G, H)** catalase; **(I, J)** sucrase; **(A, C, E, G, I)** 0-10cm soil layer. **(B, D, F, H, G)** 10-20cm soil layer. The bars show the standard errors. Lowercase letters represent the significant difference within the same ecological regions under different cropping systems, while uppercase letters indicate the significant difference within different ecological regions under the same cropping systems. Significance was employed at 0.05.

The influence of the cropping system on soil enzyme activity in different ecological regions is listed in **Table 6**. In the 0-10 cm and 10-20 cm soil layers, the NR/ALPT/UE/CAT/SC contents were significantly affected by the cropping system (*P* < 0.01), ecological region (*P* < 0.01), and their interaction (*P* < 0.01). The exception was the ALPT/UE enzyme activity, which was not significantly affected by the interaction of the cropping system and the ecological region in the 0-10 cm soil layer. The effects of different cropping systems on the soil enzyme activity in the ecological regions are shown in **Figure 4**. The soil enzyme activity decreased significantly with the soil depth (*P* < 0.05). Meanwhile, the range of the soil enzyme activity was higher in the 0–10 cm soil layer than in the 10–20 cm soil layer for all cropping systems in different ecological regions (**Figure 3**). There were significant differences in the soil enzyme activity between different ecological regions (*P* < 0.05). In the 0-10 cm and 10-20 cm soil layers, the ranking of the regions based on the soil enzyme activity was MY>GN>HZ. Meanwhile, the order of the cropping systems based on the UE, NR, and SC activities was mixed crops > legumes > oat (**Figure 4**).

**Table 6:** Variance analysis of soil enzyme activity in different cropping systems in different ecological regions.

Soil depth	Source of variation	UE	ALPT	NR	CAT	SC
0-10	District	9.33**	89.26**	63.97**	50.51**	3.27**
	Treatment	28.14**	24.78**	86.68**	30.09**	50.19**
	D * T	1.35	1.06	2.88**	3.77**	6.32**
10-20	District	23.99**	38.66**	102.28**	64.06**	43.83**
	Treatment	10.87**	9.41**	50.84**	10.09**	5.88**
	D * T	3.21**	3.63**	10.2**	12.63**	6.29**

UE, urease; ALPT, alkaline protease; NR, nitrate reductase; CAT, catalase; SC, sucrase. * and ** represent significant differences at the 0.05 and 0.01 level, respectively.

In the 0-10 cm soil layer, the cropping systems with the highest NR enzyme activity in HZ, GN, and MY were YS, YC, and YS. These activities were significantly higher than those of the other cropping systems (*P* < 0.05). Meanwhile, the YC (GN) and YS (MY) were significantly higher than those of the other cropping systems in other regions (*P* < 0.05) (**Figure 4A**). The ranking of the cropping systems based on the enzyme activities of ALPT was oats > mixed crops > legumes (except for GN). YC had the highest ALPT enzyme activity in GN. It was significantly higher than that of the other cropping systems (*P* < 0.05). The activity of ALPT was the highest in MY (**Figure 4C**). The cropping systems with the highest UE enzyme activity in the HZ, GN, and MY were YS, YC, and YS. The activities were 66.44%, 53.15%, and 55.43% higher, respectively, than those of the oat monoculture (*P* < 0.05) and significantly higher than that of the other monoculture cropping systems (*P* < 0.05) (**Figure 4E**). The CAT enzyme activity was higher in the mixed crops than in the monoculture in all ecological regions. Additionally, the CAT enzyme activity of YJ was significantly different from that of other ecological regions (*P* < 0.05). The activities were significantly higher than those of the monoculture cropping systems (*P* < 0.05) (**Figure 4G**). The ranking of the cropping systems based on the SC activity was mixed crops > legumes > oats. The cropping systems with the highest SC enzyme activity in the HZ, GN, and MY regions were YJ, YC, and YC, respectively (*P* < 0.05) (**Figure 4I**).

In the 10-20 cm soil layer, in the GN, the NR activities were significantly higher in YS and YJ than in other cropping systems (*P* < 0.05). The highest NR activity in MY was 0.230 mg·kg^-1^·24^-1^ in YS (**Figure 4B**). YJ had the highest ALPT activity among all ecological regions (except for GN (YC)) (**Figure 4D**). YS had the highest UE activity (except for MY (YJ)). It was higher than that of Y (**Figure 4F**). The CAT activity was higher for mixed crops than for monocultures, except in MY. The CAT activity in HZ and GN was the highest for the YJ and YC cropping systems. It was significantly higher than that of the other cropping systems (*P* < 0.05). The CAT activity in MY was the highest in the C cropping system. However, the CAT activity of YC was significantly lower than that of the other cropping systems (*P* < 0.05) (**Figure 4H**). The cropping systems with the highest SC activities in the HZ, GN, and MY regions were YC, YC, and C, respectively. They were 39.86%, 50.63%, and 23.92% higher, respectively than that of the oat monoculture (**Figure 4J**).

### 3.5 soil microbial community

**Figure 5:**
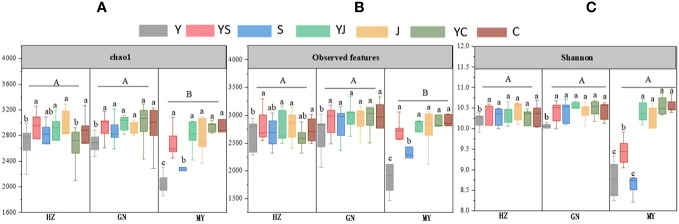
Abundance and diversity indices of bacterial microbial communities in soil samples from different ecological regions with different cropping systems. **(A)** chao1. **(B)** observed features. **(C)** Shannon. Different lowercase letters represent significant differences between cropping systems in the same ecological regions, and different uppercase letters represent significant differences between ecological regions.

**Figure 6:**
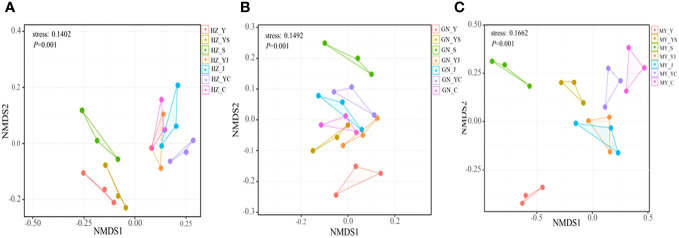
Non-metric multidimensional scaling (NMDS) analysis of bacterial OTU levels in different ecological regions with different cropping systems. **(A)** Relative differences in bacterial community composition in HZ. **(B)** Relative differences in bacterial community composition in GN. **(C)** Relative differences in bacterial community composition in MY.

**Figure 7:**
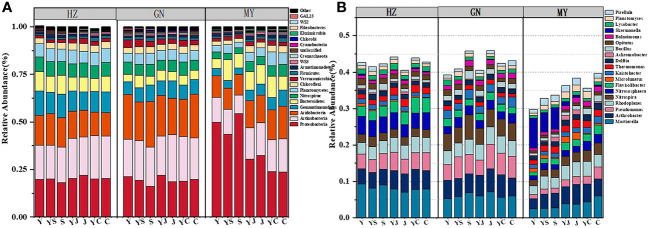
Relative abundance of bacterial phyla in different ecological regions under different cropping systems **(A)**. relative abundance of bacterial genera in different ecological regions under different cropping patterns **(B)**.

The results of one-way ANOVA showed that mixed cropping significantly increased Chao1 abundance and observed species values of bacteria as well as Shannon diversity in all ecological regions compared to oat unicast (*P* < 0.05). Chao1 abundance and observed species values were significantly higher in HZ and GN than in MY, while Shannon diversity did not differ significantly among the three regions (**Figure 5**). NMDS analysis revealed that there were differences in the bacterial communities of different cropping systems in different ecological regions (**Figure 6**). At the level of bacterial phylum, Proteobacteria, Actinobacteria, Acidobacteria, Gemmatimonadetes, Bacteroidetes, Nitrospirae, Chloroflexi, and Planctomycetes were found to be in soil bacterial communities dominated (**Figure 7A**). Meanwhile, the relative abundance of phylum levels in the bacterial community was higher in MY than in HZ and GN. In addition, the relative abundance of Nitrospirae was higher in legume monoculture and mixed sowing than in oat monoculture (**Figure 7A**). At the bacterial genus level, the soil bacterial community was found to be dominated by the Ralstonia, Arthrobacter, Pseudomonas, Rhodoplanes Nitrospira, Nitrososphaera genera dominated the soil bacterial community (**Figure 7B**). Meanwhile, the relative abundance of dominant bacterial communities at genus level was higher in HZ and GN than in MY. In addition, the relative abundance of bacterial communities at the genus level was higher in the mixed-seeded soil than in the oat monoculture, especially the relative abundance of Nitrospira and Nitrososphaera bacteria (**Figure 7B**).

### 3.6 Key factors affecting forage yield

**Figure 8:**
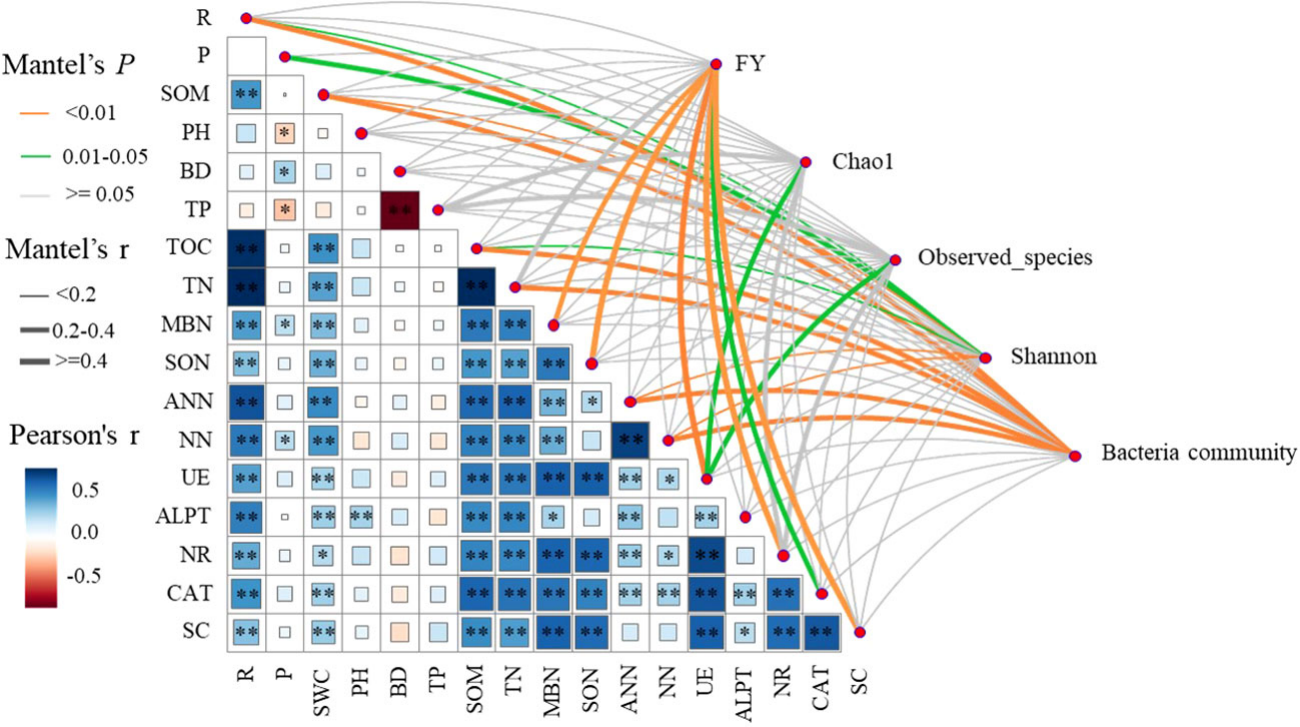
Correlation of forage yield (FY), microbial community and diversity with cropping systems (P), ecological regions (R), soil physical properties, soil nitrogen fractions, and soil enzyme activities. width of the Mantel edge corresponds to the Mantel r value, and the edge color indicates statistical significance. ∗ denotes 0.01 < *P* < 0.05, ∗∗ denotes *P* < 0.01, and ∗∗ denotes *P* < 0.001.

Pearson’s correlation analysis showed that ecological regions (R) were significantly (P < 0.05) positively correlated with SWC, SOM, TN, MBN, SON, ANN, NN, UE, ALPT, NR, CAT, and SC. Cropping systems (P) was significantly (*P* < 0.05) positively correlated with BD, MBN, and NN, and significantly (*P* < 0.05) negatively correlated with PH and TP. The results of Mantel test analysis showed that MBN, SON, UE, CAT, and SC were significantly (*P* < 0.05) positively correlated with forage yield. UE was significantly (*P* < 0.05) positively correlated with Chao1 and observed number. ANN, NN, SOM, P, and R were significantly (*P* < 0.05) positively correlated with Shannon. ANN, NN, SOM, TN, SWC, R and bacteria communities were significantly positively correlated (*P* < 0.05) (**Figure 8**).

**Figure 9:**
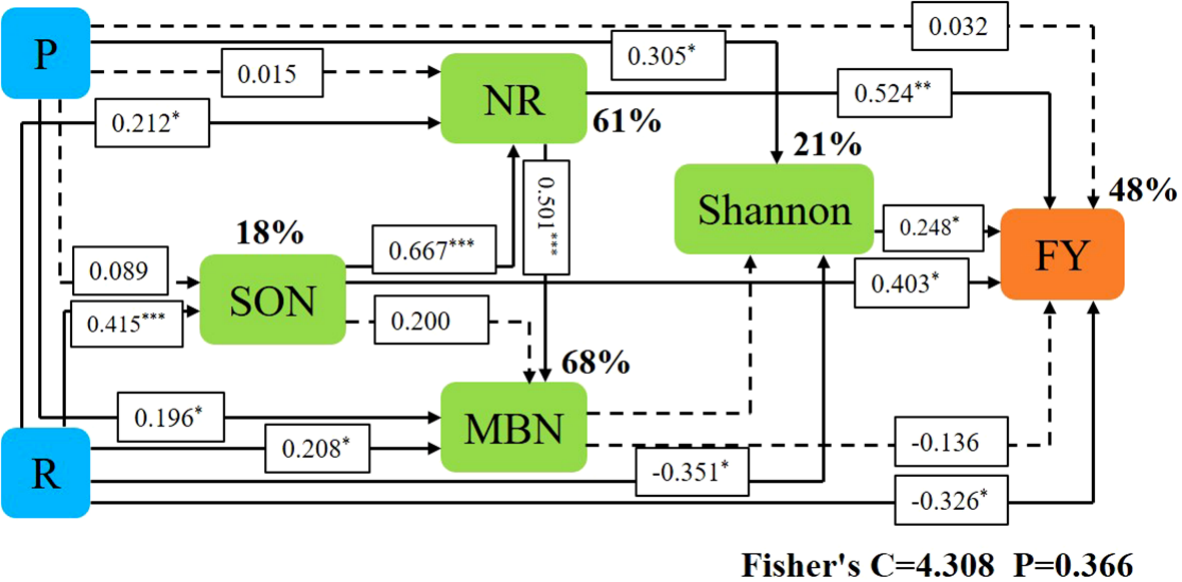
Piecewise structural equation modeling (SEM) describing the effect of cropping systems on forage yield in different ecological regions. Effects of soil enzyme activity (NR), soil nitrogen fractions (SON, MBN), soil microorganisms (Shannon), cropping pattern (R), and S ecoregion (P) on forage yield (FY). Solid lines indicate significant effects and dashed lines indicate non-significant effects. *, ** and *** represent significant differences at 0.05, 0.01 and 0.001 levels, respectively.

Variables in Mental that had a significant effect on forage yield and microbial community composition were selected as predictors to generate slice SEM to identify key drivers of forage yield. For forage yield, path analysis indicated that NR, Shannon, and SON had significant direct positive effects on FY. Cropping systems increased forage yield by increasing SON, which in turn increased NR and ultimately increased forage yield. Ecological regions increased forage yield by increasing Shannon and thus increasing forage yield (**Figure 9**).

## 4 Discussion

Soil quality is directly affected by alterations in biological, biochemical, microbiological, physical, and chemical factors. The microbiological activities of soil can affect soil fertility and plant growth because they speed up the cycling of nutrients, hormones, and enzymes required by plants for growth and development. Different agricultural management practices can influence enzyme activities and microbial biomass by altering the soil microclimate, soil microorganism habitat, and nutrient cycling (Lozano et al., 2017). Reasonable farming methods (Gao and Xie, 2023) and planting patterns (Spiegel et al., 2007) can improve resource utilization and result in increased yields. The cropping systems ultimately contribute to crop growth and development and yields (Vines et al., 2006) by influencing soil physicochemistry (Xiong et al., 2021), soil nutrients, soil enzyme activity in the cultivated layer (Khatiwada et al., 2020), and the soil microbial system (Bai et al., 2022).

### 4.1 Effect of cropping systems on forage yield in different ecological regions

Forage yield is a critical indicator of farming practices and is typically optimized to ensure sufficient livestock feed (Kawamura et al., 2010). Many studies have analyzed the effects of legume incorporation in cropping systems on plant biomass and aboveground productivity (Sturludóttir et al., 2014; Halim et al., 2016). The effect of legumes on aboveground biomass has been studied from the aspects of interspecific relationships and symbiotic nitrogen fixation (Karpenstein-Machan and Stuelpnagel, 2000; Khatiwada et al., 2020). It has been shown that when the land equivalent ratio (LER) of a legume-grass mixture is greater than 1, the mixture utilizes plant growth resources more efficiently than a pure crop, resulting in higher aboveground biomass and productivity (Scheffer et al., 1990). It is typically assumed that N addition always improves forage yield. However, the lack of legumes in cropping systems and the addition of nitrogen fertilizers, may reduce microbial biomass, adversely affecting the soil microflora (Deforest et al., 2004). In addition, the risk of nitrate leaching is increased (Inoue et al., 2021); thus, incorporating perennial legumes in rangeland planting is critical. Legumes fix atmospheric N through the symbiotic relationship with rhizobia. The amount of atmospheric N fixed in grass–legume pastures has been estimated at > 500 kg N·ha ^−1^·yr ^−1^ (Lüscher et al., 2014). A large fraction (70-80%) of the fixed N is transferred to the crop, increasing grassland productivity and the economic and ecological potential (G Carlsson, 2003). In the present study, the YS, YJ, and YC cropping systems produce higher forage yields than the monocultures in different ecological zones. The reason is that organic matter, soil TN, NN, MBN, CAT and UE activities, and other enzyme activities are critical for enhancing nutrient uptake by plants. Moreover, the inclusion of legumes in the mixture increases the N amount supplied to grasses due to their N-fixing ability, improving the growth and development of grasses (Tahir et al., 2022). Our results are consistent with those of a study on the N yield of a grass-legume mixture. It showed 40%–60% of the legumes in the grass–legume mixtures efficiently transformed N into biomass by stimulating the N uptake from symbiotic and nonsymbiotic sources (Nyfeler et al., 2011). In addition, the advantages of legume-grass mixtures is attributed to different aboveground and belowground growth habits and morphological characteristics of the crops and the higher utilization efficiency of water and solar radiation. Some studies have shown that an oat-legume mixture increased the resistance to lodging and pests. The legume vines are supported by the oat stalks, resulting in higher forage yields (Cai et al., 2019). This study also found that the cropping system with the highest forage yield differed in different ecological zones (HZ (YS), GN (YC), and MY (YS)) and the forage yield decreased with the elevation. The likely reason is that environmental factors, such as the altitudinal gradient, light, temperature, rainfall, and soil nutrients differ in different ecological zones, affecting crop growth and development and yield (Rosbakh et al., 2017). As the elevation increases, the cumulative temperature decreases, the crop’s reproductive period lengthens, and the plant height and spike length shorten, resulting in a small reduction in forage yields (Suter et al., 2015). An increase in altitude alters the microclimate of the legume-grass community due to differences in crop types and their adaptability to the environment (Waigwa et al., 2020).

### 4.2 Effect of cropping systems on soil physical properties in different ecological regions

Cropping systems affect soil physicochemical properties, but the impact on soil properties and soil quality, among others, varies due to different climatic conditions and soil types (Wu et al., 2021; Tahir et al., 2023). Cropping systems can improve the carbon and nitrogen concentrations, physicochemical traits, bulk density, and soil pH (Bedoussac and Justes, 2010; Tongjin et al., 2014; Sen and Fengzhi, 2018; Cuartero et al., 2022). However, reductions in soil organic carbon and soil quality due to inappropriate or excessive tillage or inappropriate fertilizer use can exacerbate soil degradation and reduce soil porosity, water conductivity, effective water content, and crop growth (Yang et al., 2023). In this study, the mixed crops had higher SOM and TP and lower pH and BD than the oat monoculture in all ecological zones, and there was no significant effect on TP and BD in the 0-10 cm layer. The reason is that oats and the three legumes are short-rooted crops; the roots are predominantly located in the 0-10 cm soil layer. Their ability to improve the surface soil BD and TP role is significant because the fibrous root system of oats is well-developed and dense, resulting in a granular soil structure that improves the permeability of the upper soil layer. As a result, the plants obtain sufficient water. The intense competition for water exacerbates the legume-grass root plasticity during periods of water scarcity to achieve the mechanism of grass and bean symbiosis. Thus, the legume-grass symbiosis mechanism is achieved (Westhoek et al., 2021). The SOM content in the legume-grass mixtures was higher in the 0-10 cm layer than in the 10-20 cm layer in all ecological zones, demonstrating the advantage of the legume-grass mixture over the oat monoculture. The highest SOM content occurred in the YS system. In most of the legume-grass mixtures, the soil nutrient content decreased from the surface layer to the lower layer, and the fluctuations were higher in the surface layer than in the lower layers. This result was attributed to the high permeability of the surface soil layer, coupled with the rapid turnover of the surface litter and residue, increasing the conversion of humus and decreasing the decomposition of organic matter. The microorganisms in the soil were more active in the root layer due to the higher nitrogen fixation capacity of forage peas, promoting the decomposition of humus and resulting in a higher SOM content (Reinhart, 2013; Neugschwandtner et al., 2023). In contrast, there were large differences in the SOM content between the ecological zones due to differences in parent soils. The ranking of the regions based on the SOM content was MY>HZ>GN, but the ranking based on SOM range of variation between regions was GN>HZ>MY. This inconsistency was attributed to GN having the highest altitudinal gradient and the lowest temperature. Low temperatures reduce the rate of organic matter decomposition. The high-altitude zones showed higher fluctuations in SOM content (Waigwa et al., 2020).

### 4.3 Effect of cropping systems on soil nitrogen fractions in different ecological regions

Nitrogen exists primarily in organic form in the soil, accounting for about 85%-90% of the total soil nitrogen. Organic nitrogen can only be absorbed and utilized by plants through mineralization into inorganic nitrogen (Schulten and Schnitzer, 1997; Thomas et al., 2016; Shahid et al., 2017). Although some soils can mineralize sufficient N to satisfy the growth requirements of high-yielding crops(Peoples et al., 2004; Ladha et al., 2022), additional reactive N produced biologically or chemically is generally needed to ensure agricultural productivity. Legumes strongly contribute to the diversification, sustainability, and long-term productivity of agricultural systems. Their ability to fix atmospheric N through symbiosis with N-fixing soil bacteria ensures that plants obtain sufficient N (Kessel and Hartley, 2000). In this study, the TN/ANN/NN contents were significantly influenced by the ecological zone and cropping system. The ranking of the cropping systems based on the TN content in all ecological zones was mixed crops > legume monoculture > oat monoculture, whereas that based on the ANN and NN contents was legume monoculture > mixed crops > oat monoculture (Tahir et al., 2023). Meanwhile, the YS cropping system exhibited the highest TN content in HZ and MY, this result was attributed to the stronger ability of forage peas to fix nitrogen than common vetch and fava beans. However, the YC system had the highest TN content in GN, this may be the altitude increased and the temperature decreased, the ability of forage peas to cope with fix nitrogen decreased. Nodulation was inhibited when the temperature was lower than the maximum threshold, but the nitrogen fixation ability of fava beans was not affected because of their thick stalks and leaves, which are adapted to the low temperature. The soil TN content of the legume-oat mixture was the highest (Kage, 1997; Neugschwandtner et al., 2023). In this study, the ranking of the cropping systems based on the MBN and SON contents in all ecological zones was mixed crops > legume monoculture > oat monoculture, and the highest contents occurred in HZ (YS), MY (YS), and GN (YC). This ranking was the same as that based on the SOM content. The reason is the high correlation between the contents of MBN and SOM. MBN derived from SOM and occur in plant and animal residues. Mixing oats with legumes increases the abundance and activity of soil microorganisms due to legume-specific rhizobacteria, resulting in increased decomposition of organic matter. The SON is utilized by soil microorganisms, increasing soil fertility, promoting plant uptake, and increasing aboveground biomass. However, the nitrogen fraction content of the topsoil decreased with the soil depth, and stratification occurred, probably due to long-term soil tillage and the spatial distribution of the crop root system (Gwenzi et al., 2009; Liu et al., 2022)

### 4.4 Effect of cropping systems on soil enzyme activities in different ecological regions

Soil enzyme activity is a vital indicator of organic matter decomposition since nutrients are cycled in the soil (Nannipieri et al., 2012; Hussain et al., 2021). Soil enzymatic activity is affected by the physicochemical traits and microbial communities of the soil (Gu et al., 2009; Latati et al., 2014). Soil enzyme activities are crucial for recycling nutrients, decomposing organic matter, and influencing microbial functions (Sinsabaugh et al., 2008). Our research shows that the activities of five enzymes decreased with the soil depth, consistent with the soil nutrient trend. This result was attributed to the surface soil layer is exposed to temperature and humidity fluctuations, the microbial activity is higher in this layer, and growth and reproduction occur faster. This is consistent with previous research (Zhou, 2012).

NR is a regulatory and rate-limiting enzyme used for nitrate assimilation in plants. It is sensitive to the nitrogen concentration and indirectly affects plant nitrogen uptake and utilization (Pu et al., 2019). NR is the key enzyme in denitrification, reducing nitrate and nitrite to NO, N_2_O, and N_2_ under anoxic conditions. This process typically leads to nitrogen loss of agricultural soils and emissions of the greenhouse gas N_2_O (Chen et al., 2012). Mineralization, nitrification, and denitrification are key biochemical processes that affect nitrogen utilization, loss, and greenhouse gas emissions during nitrogen metabolism (Shcherbak et al., 2014; Junli et al., 2018). Therefore, factors affecting soil nitrogen metabolism also influence enzyme activities in different cropping systems. Mixed grass-legume crops influence soil properties and soil microbial composition and abundance, in turn affecting soil enzyme activities. In our experiment, the NR activity was higher in mixed crops than in monocultures in all ecological zones because the enzyme activity was correlated with the content of soil NN, which was the substrate for the redox reaction. Therefore, the soil NN and ANN contents were higher in the mixed crops, in agreement with the findings of Weng et al. (Weng et al., 2013) Furthermore, the cropping patterns with the highest NN activity were not the same in different ecological zones (HZ (YS), GN (YC), and MY (YS)). We attribute this phenomenon to the different adaptations of beans to the environment of different ecological zones, resulting in different types and qualities of rhizomatous exudates, depending on the type and total amount of nitrogen fixation. Previous research revealed that alfalfa root exudates positively influenced enzyme activity and increased the soil microbial population and nitrogen requirements (Moreau et al., 2019), providing a plausible explanation for the higher NR activities in this study. In the soil nitrogen cycle, ALPT hydrolyzes proteins into oligopeptides and amino acids, a rate-limiting process of soil nitrogen mineralization (Madejon and Ciadamidaro, 2014). The ranking of the crop systems based on the ALPT activity in all ecological zones was oat monoculture > mixed crops > legume monoculture. This finding can be attributed to the nitrogen uptake preference of legumes. ANN and NN are present in the soil solution as AN and ANN until they are absorbed and utilized by legumes (Xu et al., 2019). Furthermore, the plant nitrogen uptake preference indirectly influences the carbon and nitrogen availability through the effects on root exudates and microbial biomass (Greenfield et al., 2020). There was no need for microorganisms to release nitrogen through decomposing organic matter by mineralization (He et al., 2021); thus, the ALPT activity was significantly reduced. UE is a hydrolase with a crucial effect on the soil nitrogen cycle. It transforms small nitrogen-containing organic substrates into inorganic nitrogen-containing compounds (such as ammonia) to provide available nitrogen for plant growth and development (Cordero et al., 2019). In this study, the ranking of the crop systems based on the UE activity was mixed crops > legume monoculture > oat monoculture in all ecological regions. Meanwhile, the legume–grass mixtures had higher UE activity than the monocultures. This advantageous effect of mixed crops on UE activity can be attributed to an increase in the microbial population and the release of a greater proportion of nitrogenous substances (ANN and NN) in the root exudates, increasing UE activity and providing nutrients for plant uptake. The UE activity is highly dependent on the availability of nitrogen for nitrogen-cycling enzymes, resulting in increased enzyme activity. A positive correlation was observed between the nitrogen content and the UE activity (Ibrahim et al., 2020). CAT promotes the decomposition of hydrogen peroxide, a free radical harmful to terrestrial plants when it enter the water. It prevents the toxic effect of hydrogen peroxide on soil enzymes (Lemanowicz, 2019). It acts as an important oxidoreductase system for the synthesis of humus in soil (Purev et al., 2014). CAT plays an important role in soil ecosystems and has been used as a biological activity index to evaluate soil quality (Qu et al., 2020). The CAT activity was the highest enzyme activity in all ecological regions (HZ (YS), GN (YC), and MY (YS)). The activity was significantly higher in the mixed crops than in the monocultures. High activity indicates high soil fertility and the presence of aerobic microorganisms in the soil (Tiwari, 1987). The CAT activity depends on the soil type and crop (Filipek-Mazur et al., 2020). Therefore, it varied in different ecological regions. The highest CAT activity at the lowest altitude in MY may be due to the higher temperature in this region. A high activity ensures the survival of aerobic microorganisms.

SC hydrolyzes sucrose and reflects the convertibility of soil organic carbon (Cantarella et al., 2018). In the present study, the SC activity was higher in mixed legume-grass systems than in monocultures. In addition, the cropping systems with the highest SC enzyme activities in the HZ, GN, and MY regions were YJ (13.65 mg·kg^-1^·24^-1^), YC (15.78 mg·kg^-1^·24^-1^), and YC (16.83 mg·kg^-1^·24^-1^), respectively. The likely reason is the nitrogen fixation ability of the legumes, resulting in increased root secretion and root decomposition, increasing the SC activity.

### 4.5 Effect of cropping systems on soil microbial community in different ecological regions

Soil microorganisms, as indicators of soil health, are involved in nutrient cycling (Crecchio et al., 2004) and transformation processes and can be sensitive to changes in soil (Song-Juan et al., 2015). Mixed seeding can directly affect soil microbial community structure by altering soil nutrient availability. In the present study, there were differences in soil bacterial community structure and diversity among the planting patterns in different ecological zones. We found that soil bacterial a-diversity was significantly higher in mixed cropping than in oat monocropping, and the mixed cropping patterns with the highest bacterial a-diversity were not the same in different ecological zones. It indicates that nitrogen-fixing microorganisms specific to legumes increase soil microbial diversity (Dommergues, 1987; Murali et al., 2012). At the same time, differences in the biological nitrogen fixation capacity of leguminous crops in different ecological regions make soil nitrogen cycling and utilization different, indirectly affecting soil bacterial microbial diversity(Kennedy and Tchan, 1992; Rahman, 2013). In addition, we found that the bacterial a-diversity was significantly higher in HZ and GN than in MY, possibly due to different initial microbial communities and different responses of soil microbes in different ecological zones to cropping patterns (Liu et al., 2023). We also found that Proteobacteria were the most abundant phylum in the studied flora in different ecological zones, followed by Actinobacteria, which is in line with previous findings (Jing et al., 2018). The high environmental adaptability, rapid growth and reproduction, and high substrate uptake capacity of Proteobacteria are the main reasons for the dominance of Ascomycetes in soils of different ecological zones of the alpine (Salcher et al., 2010). And Actinobacteria can decompose cellulose, lignin and other organic matter, promote the formation of soil agglomerate structure, and improve the quality of the soil, therefore, the number of actinomycetes in the mixed planting soil of graminaceous and leguminous is increased (Li et al., 2020). At the genus level, Sulfurifustis was the dominant genus. The differences in dominant genera among different planting patterns in the same ecological zone were not significant, but the planting patterns had a significant effect on some low abundance nitrogen transformation (Nitrospira, Nitrososphaera) related genera, and the responses of nitrogen transformation microorganisms in soils of different ecological zones planted in different planting patterns were not the same. It indicates that planting a mixture of grasses and legumes in different ecological zones of the alpine can increase the abundance of species related to soil nitrogen cycling and improve soil nitrogen utilization and cycling. Soil bacterial b-diversity varies between cropping patterns. It is due to the fact that bacterial b-diversity is mainly driven by plant diversity and dominant species of bacteria (Liu et al., 2023). Whereas mixed seeding increases plant diversity, different plant characteristics and soil environments produce different ecological niches (Hamid et al., 2021), which in turn affect specific soil microbial communities. Differences in the composition and amount of root secretions of different species can lead to alterations in the metabolism of soil microorganisms, which in turn affects the composition of soil microorganisms (Nayyar et al., 2010). In addition to this, different plant communities have different apoplasts, which can affect the living environment of soil microorganisms and indirectly influence the composition of soil microbial communities (Liu et al., 2023).

### 4.6 Key factors affecting forage yield and microbial communities

Correlation between soil physicochemical properties and enzyme activities with soil microbial community structure and forage yield (Liu et al., 2017; Tahir et al., 2023). It has been shown that soil bacterial communities correlate with all physicochemical properties of soil, with soil pH and total nitrogen having the most significant effects on bacterial communities (Hester and Sloey, 2016). Li et al. showed that soil pH, SOM, and bulk weight helped to explain the changes in soil microbial composition observed in the study, while changes in soil quick nutrients (N-NO_3_^-^, AK, and AP) had a significant effect on bacterial abundance and diversity (Li et al., 2022). Soil enzyme activities are closely related to bacterial and fungal taxa (Aon and Colaneri, 2001). The resource allocation model of extracellular enzyme activity states that soil microorganisms mineralize and recycle deficient soil nutrients through the release of extracellular enzymes (Sinsabaugh and Moorhead, 1994), which highlights the important role of soil microorganisms in soil nutrient cycling and overall soil health. It is generally believed that the activities of urease, protease, phosphatase and cellulase are closely related to the microbial biomass, which will increase with the increase of microbial biomass, and can characterize the cycling status of soil carbon, nitrogen and phosphorus nutrients(Tiwari et al., 1989; Mersi and Schinner, 1991). The results of Mantel test analysis showed that MBN, SON, UE, CAT, and SC were significantly (P < 0.05) positively correlated with forage yield. UE was significantly (P < 0.05) positively correlated with Chao1 and observed number. ANN, NN, TOC, P, and R were significantly (P < 0.05) positively correlated with Shannon. ANN, NN, SOM, TN, SWC, R and bacteria communities were significantly positively correlated (P < 0.05). This suggests that enzyme activities and soil N fractions contribute significantly to forage yield and microbes. Soil SON, MBN, NR and microbial diversity were the main drivers of increased forage yield in different ecoregional cropping patterns, which is consistent with the results of several studies under different cropping systems (Afzal et al., 2005; Tahir et al., 2023). The reason for this could be: (1) Mixed cropping increases species diversity, has complementary ecological niche advantages, more balanced nutrient distribution, and increased forage production (Stephan and Schmid, 2000; Naeem et al., 2009). (2) The characteristic rhizomatous and biological nitrogen fixation of legume crops promotes soil nitrogen cycling and utilization, increases soil nutrients, and improves nitrogen-related soil enzyme activities. (3) Feedback regulation by soil microbes. Nitrogen-fixing bacteria produced by legume nodules, which shifts the soil microbial community from one dominant species to another (Ben-Chuan et al., 2022), increase soil microbial abundance, such as nitrifying bacteria, which act on nitrogen cycling and utilization, which is taken up and utilized by plants, and which microorganisms use to influence nutrient cycling and plant growth through a feedback regulation effect (Kuypers et al., 2018).

## 5 Conclusion

This study showed that legume-grass mixtures resulted in higher forage yields, soil organic matter, nitrogen fractions, enzyme activities, bacterial community-α diversity and relative abundance of dominant bacteria than monocultures in all ecological regions, improving the soil nutrient status. The soil physical property indicators, nitrogen fractions, and enzyme activities had larger ranges in the 0-10 cm soil layer than in the 10-20 cm layer, with larger values in the surface layer than in the subsurface layer. Based on the content of SOM, TN, MBN, ALPT and SC and the relative abundance of phylum levels in the bacterial community, the ecological regions were ranked as MY>GN>HZ. The forage yield decreased with increasing altitude: GN (3203 m; YH 8.89 t·ha^-1^) < HZ (2661 m; YH 9.38 t·ha^-1^) < MY (2513 m; YH 9.78 t·ha^-1^). The highest forage yields were as follows: MY (YS, 13.00 t·ha^-1^), GN (YC, 10.59 t·ha^-1^), and HZ (YS,10.63 t·ha^-1^). Based on the results, we recommend planting oast and forage peas in the Qilian mountain basin, oats and fava beans in the Sanjiangyuan District, and oats and forage peas in the Huangshui valley. Our results provide guidance for eco-friendly, sustainable, and cost-effective forage production in the Qinghai Alpine Region of China.

## Data availability statement

The original contributions presented in the study are included in the article/supplementary material. Further inquiries can be directed to the corresponding author.

## Author contributions

FL: Conceptualization, Methodology, Visualization, Writing – original draft. WHL: Funding acquisition, Writing – review & editing. WM: Software, Writing – review & editing. XM: Writing – review & editing, Data curation. KL: Writing – review & editing, Software. ZJ: Writing – review & editing, Formal Analysis. WL: Writing – review & editing, Data curation.
